# Cross-cultural adaptation and validation of the Chinese version of the revised surveys on patient safety culture™ (SOPS®) hospital survey 2.0

**DOI:** 10.1186/s12912-022-01142-3

**Published:** 2022-12-26

**Authors:** Yinghui Wu, Wenzhe Hua, Daqiao Zhu, Ryo Onishi, Yanna Yang, Tomonori Hasegawa

**Affiliations:** 1 School of Nursing, Shanghai Jiao Tong University, 227 S Chongqing Rd, Shanghai, 200025 China; 2 Department of Social Medicine, Toho University School of Medicine, 5-21-16 Omori-Nishi, Ota-ku, Tokyo, 143-8540 Japan

**Keywords:** Safety culture, Patient safety, Nurse, Reliability, Validity

## Abstract

**Background:**

Surveys on Patient Safety Culture™ (SOPS®) Hospital Survey (HSOPS 1.0), developed by the U.S. Agency for Healthcare Research and Quality in 2004, has been widely adopted in the United States and internationally. An updated version, the SOPS Hospital Survey 2.0 (HSOPS 2.0), released in 2019, has not yet been applied in China. The aim of the present study was to translate HSOPS 2.0 into Chinese version with cross-cultural adaptations and test its psychometric properties.

**Methods:**

A convenience sample was used. Hospital nurses (*N* = 1013) and a sub-set (*n* = 200) was invited for the re-test. A three-stage study was conducted. Firstly, the HSOPS 2.0 was translated by a panel. Secondly, the content validity was tested using the two-round Delphi method and cognitive interview. Next, the construct validity was tested by the confirmatory factor analysis and further demonstrated by the convergent validity, discriminant validity, and correlations with the outcome of patient safety. Thirdly, the reliability was tested by internal consistency reliability and re-test reliability.

**Results:**

The “float or PRN” and “manager” words were deleted as considered unfitted for the Chinese health care system. The content validity index provided evidence of strong content validity (I-CVI = 0.84 ~ 1.00, S-CVI = 0.98). Confirmatory factor analysis revealed a good model fit (χ^2^/df = 4.05, RMSEA = 0.06, CFI = 0.94) and acceptable factor loadings (0.41 ~ 0.97). Convergent validity, and discriminant validity supported the factorial structure of the Chinese version of HSOPS 2.0. Further evidence for the construct validity was derived from correlations with the outcome of patient safety (*r* = 0.10 ~ 0.41). A good internal consistency (Cronbach’s *α* = 0.68 ~ 0.93, McDonald’s omega = 0.84 ~ 0.96) and test-retest reliability (ICC = 0.78 ~ 0.95) showed acceptable reliability. Additionally, Chinese nurses reported markedly lower scores for three dimensions, including “Response to Error”, “Communication Openness”, and “Reporting Patient Safety Events”, when comparing the findings of this study with those from U.S. research utilizing the HSOPS 2.0.

**Conclusion:**

The Chinese version of HSOPS 2.0 demonstrated good validity and reliability in a Chinese sample of hospital nurses, which suggests that it can be used to measure nurse-perceived patient safety culture in future research and practice. Psychometric properties of the Chinese version of HSOPS 2.0 among other Chinese healthcare professionals remain to be confirmed.

**Supplementary Information:**

The online version contains supplementary material available at 10.1186/s12912-022-01142-3.

## Background

Although the quality of healthcare is increasing on the global level, patient safety is still a major concern for policymakers [1, 2]. According to the World Health Organization, patient safety can be achieved by preventing and reducing risks, errors, and harm that occur to patients while providing health care [3]. Patient safety culture (PSC) is defined as the common attitude, beliefs, values, and behaviors of health caregivers shared in the process of ensuring patient safety [4], which has an important role in reducing the occurrence of adverse events and benefiting staff well-being [5, 6]. Several empirical studies have shown a correlation between PSC and job satisfaction, burnout, and workplace violence [7, 8], which in turn affects patient safety. For example, during the COVID-19 pandemic, a significant increase in melancholy, anxiety, and uncertainty was observed among all medical professions [9], yet, health professionals who maintained higher PSC levels had greater levels of resilience [10]. Therefore, it is also of crucial importance to assess and comprehend the perceptions, attitudes, norms, and values of patient safety and its thresholds to ensure the maximum degree of PSC in the healthcare institution [11].

Survey questionnaires are one of the most popular methods for assessing hospital PSC. This method provides a clear picture of the current hospital PSC, highlights its strengths, and pinpoints particular issues that impede patient safety improvement [12]. It can also set a standard and improve PSC measures across time and between organizations at national and international levels [13]. The Hospital Patient Safety Culture Survey (HSOPS) is one of the most appropriate tools in terms of its psychometric properties [14]. HSOPS has been developed by the U.S. Agency for Healthcare Research and Quality (AHRQ). The HSOPS 1.0, which was released in 2004, contains 42 items across 12 dimensions, has been converted into 43 public languages and distributed to 95 nations [15]. An updated version, HSOPS 2.0, was published in 2019, comprising 32 items across 10 dimensions [16].

In China, HSOPS 1.0 has been in circulation since 2009; yet, only after 2012 the frame was adjusted to suit the needs of the Chinese health system, i.e., two dimensions (Frequency of Events Reported and Handoffs and Transitions) with 13 items were excluded [17], however, the psychometric properties were not identified for all dimensions of the Chinese version of HSOPS 1.0. In 2013, another study revealed that these 10 dimensions had low internal consistency values with Cronbach’s α < 0.7, ranging from 0.40 to 0.64 [18], and this showed inadequate reliability. Notably, these items that were deleted or contributed to low internal consistency have been dropped or reworded in the newly released HSOPS 2.0. Although HSOPS 2.0 assesses many of the same areas of PSC as HSOPS 1.0, substantial changes were made [16], and from June 2022, HSOPS 1.0 data are no longer be accepted in the Database [19]. Since there is no published Chinese version of HSOPS 2.0, we consulted the AHRQ and decided to translate HSOPS 2.0 into Chinese. Thus, the primary aim of this study was to examine the psychometric properties of the Chinese version of HSOPS 2.0, for its use in Chinese hospital settings. The secondary aim was to understand the current status of PSC in Chinese hospitals.

## Method

### Design

**Fig. 1:**
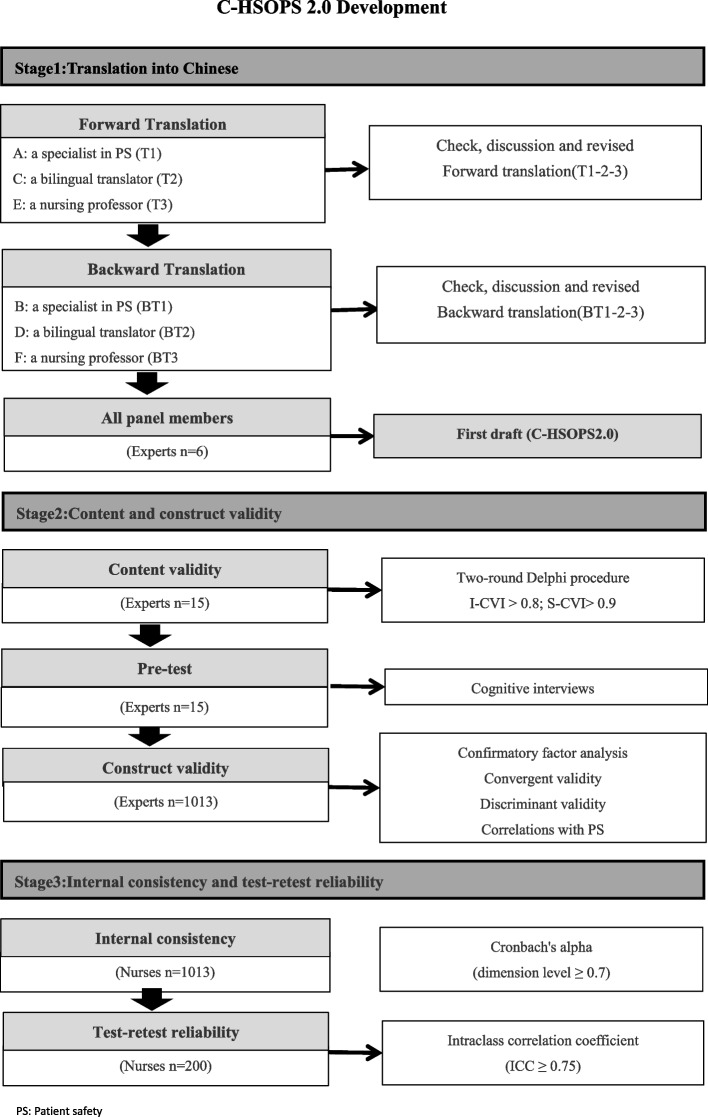
Flow diagram of overview three-stage study

A three-stage study was conducted (Fig. 1): (1) the HSOPS 2.0 was translated into the Chinese version by a panel; (2) the validity was tested by content validity and construct validity; (3) the reliability was tested by internal consistency reliability and re-test reliability.

### Instrument

The HSOPS 2.0 is shorter than HSOPS 1.0. Twenty-one items of HSOPS 1.0 were dropped, 25 items were reworded, or response options were changed, and 10 new items were added to HSOPS 2.0 [16]. The number of dimensions was reduced from 12 to 10, and number of items was reduced from 42 to 32, as shown in Table 1.

**Table 1:** Comparison of HSOPS 1.0 and HSOPS 2.0 Dimension

	HSOPS 1.0	HSOPS 2.0	Number of HSOPS 1.0 Survey items	Number of HSOPS 2.0 Survey items
DD1	Communication Openness	Communication Openness	3	4
DD2	Feedback and Communication About Error	Communication About Error	3	3
DD3	Frequency of Events Reported	Reporting Patient Safety Events	3	2
DD4	Handoffs and Transitions	Handoffs and Information Exchange	4	3
DD5	Management Support for Patient Safety	Hospital Management Support for Patient Safety	3	3
DD6	Nonpunitive Response to Error	Response to Error	3	4
DD7	Organizational Learning – Continuous Improvement	Organizational Learning – Continuous Improvement	3	3
DD8	Staffing	Staffing and Work Pace	4	4
DD9	Supervisor/Manager Expectations and Actions Promoting Patient Safety	Supervisor or Clinical Leader Support for Patient Safety	4	3
DD10	Teamwork Within Units	Teamwork	4	3
DD11	Overall Perceptions of Patient Safety*	–	4	0
DD12	Teamwork Across Units*	–	4	0
	Total**		42	32

*DD* Dimension

*Two composite measures and associated surveyitems from HSOPS 1.0 were dropped in HSOPS 2.0

**Only the survey items that are grouped into composite measures are counted in this table— two single-item outcome measures and background questions are not included in the counts

The HSOPS 2.0 items were divided into three parts: 32-items PSC measure of 10 dimensions, with 5-point scales of agreement (from ‘strongly disagree’ to ‘strongly agree’) or frequency (from ‘never’ to ‘always’), as well as an option for “does not apply or do not know”; Two single items as outcome measures that ask respondents (1) to provide an overall rating on patient safety for their unit (from poor to excellent) and (2) how many patient safety events they have reported in the past 12 months (none, 1 to 2, 3 to 5, 6 to 10, 11 or more); Six survey items that ask respondents to provide their background characteristics (respondent’s position, unit, hospital tenure, unit/work area tenure, work hours, interaction with patients),

### Translation and testing of the questionnaire

#### Stage 1: translation

The HSOPS 2.0 was translated into Chinese (permission was obtained from AHRQ) by a panel including 2 specialists A&B in patient safety and 4 bilingual English-Chinese translators (2 nursing professors C&D with PhD degree in the U.S., a nursing professor E and a nurse F who have worked both in the U.S. and Chinese hospital). In the first step, professor A, C, and E independently translated the HOSPS 2.0 into Chinese, and then they checked the translation, discussed ambiguities and discrepancies, and revised the forward translation version 1. In the second step, professor B, D, and F independently translated the Chinese version into the English version. In the final step, all panel members discussed ambiguities and discrepancies and agreed on the translation version 2.

#### Stage 2: The testing of validity

##### Content validity

Since there were not enough bilingual participants readily accessible for a pilot test with a bilingual version of HSOPS 2.0 [20], .this study used expert’s consultation through the two-round Delphi method and a pretest using cognitive interviews [21].

##### Expert consultation

We used the two-round Delphi method among 15 patient safety experts in education and scientific research (*n* = 4), clinical nursing practice (*n* = 6), and clinical nursing management (*n* = 5). Eligibility criteria for experts were: having a master’s or PhD degree and at least 10 years of working experience in their professional field. The positive and authoritative coefficients were used as an evaluation index for experts as follows:

1. Positive coefficient: it refers to the extent of experts’ concern about the study. It was evaluated by the recovery rate of the questionnaire and the rate of an expert in providing comments. In this study, the recovery rate was 100% and the rates of providing comments for the 2 rounds were 90 and 80%, respectively.
2. The authoritative coefficient (Cr): decided by the judgment criterion (Ca) and the familiarity (Cs), as follows: Cr = (Ca + Cs) /2, the greater the Cr, the greater the degree of authority. In this study, the authoritative coefficients for the 2 rounds were 0.87.

The questionnaire was sent to the expert panel (*n* = 15) via e-mail, including 2 single items (Patient Safety Rating and Number of Events Reported) and 10 dimensions. Experts rated the items through a 4 point-Likert scale (1 = irrelevant; 2 = somewhat relevant; 3 = quite relevant; 4 = very relevant), which reflected the extent to which they thought each of the 34 questions should be included in the Chinese version of HOSPS 2.0. Each item CVI (I-CVI) score was calculated using the percentage of experts who rated the item as 3 or 4, and the scale CVI (S-CVI) was calculated by computing the mean of the I-CVI scores. I-CVI scores > 0.80 are considered acceptable, and an S-CVI score > 0.90 is considered excellent [22].

Experts could also propose other amendments that they feel necessary through providing comments or justifications for each item. Changes or adaptations to the items were made based on the consensus of the experts considering four aspects: (1) semantic equivalence, (2) idiomatic equivalence, (3) experiential equivalence, and (4) conceptual equivalence [23]. Then, the pre-final version (version 3) was formed for the pre-testing cognitive interviews [24].

##### Pre-testing

Cognitive interview is a psychologically oriented method for empirically studying the ways in which individuals mentally process and respond to survey questionnaires [25]. A pre-test was conducted using cognitive interviews with 15 nurses (had to work for at least 1 year in the current hospital and 1 month in the current department). We chose the nurses as participants in consideration of their close relationship between patients and strong perception of patient safety [26, 27]. This sample size was adequate as data saturation was reached. The nurses completed the Chinese version of HSOPS 2.0 in a quiet room, followed by face-to-face, semi-structured interviews using an interview guide. The interview guide was developed to ask the nurses’ understanding of the relevance, comprehensibility, and comprehensiveness of the questionnaire [28]. Briefly, the interviewer checked through each item and encouraged that the nurses “think aloud” in order to capture their understanding of each item, what they were considering when they chose on their responses, and what was unclear [24, 28, 29]. The interviewers included one patient safety expert and one graduate student who received training in qualitative interviews. Notes were taken during the interview. Data were analyzed via content analysis by two researchers. Minor changes in wording and grammar were made after pretesting, resulting in the final version (version 4).

### Construct validity

#### Data collection

We predicted the response rate of 70% [18]. The sample size was about 915 depending on the recommended rules that sample size have ranged from ratios of 5 to 20 cases per item and from 50 participants for simple CFA models to 500 cases [30] (following formula was used: 32 items × 20÷0.7 = 915).

Convenience sampling was conducted in five large general hospitals (tertiary hospitals) in Shanghai, China. Inclusion criteria were the following: registered nurses had to work at least 1 year in the current hospital and 1 month in the current department. Finally, 1300 nurses were recruited. The paper questionnaires were completed anonymously and returned back to a locked drop-box placed in each department from April to May, 2021. The sample size in the test-retest reliability should not be fewer than one over 10 of the total research objects, and the larger the sample size the better. The subset of the nurses (*n* = 200) completed the paper questionnaires with a test-retest interval of a month [31].

#### Data analysis

The starting point in the analysis was data clean, which was performed as follows [18]: 1) not all sections were fully completed; 2) < half items were answered, or the same answer was given to all items. The remaining missing values were imputed using the expectation–maximization (EM) algorithm [32]. The next step included reverse coding of the negatively worded items.

Descriptive statistics were computed for participant background, two single item measures, and 10 dimensions of PSC. To compare the results between our survey and the original U.S. survey, the positive response rate for items and dimensions was calculated as recommended by the tool developers [19]. The positive response include “strongly agree” or “agree”, or “always” or “most of the time” for positively worded items, and answered “strongly disagree” or “disagree”, or “never” or “rarely” for negatively worded items.

Except for using AMOS 24.0 in the confirmatory factor analysis (CFA), the rest of the data analysis was performed using the SPSS 24.0. After collecting the data, calculated value of the Bartlett test [33] of sphericity was significant (*P* < 0.05) and the Kaiser-Meyer-Olkin (KMO) [34] was > 0.60, indicating that it was suitable for factor analysis. CFA was used to test the construct validity. We used the structure model equation (SME) to analyze the factor structure of the Chinese version of HOSPS 2.0 through CFA using maximum likelihood estimation (MLE). Validation of the model required establishing an acceptable level of goodness-of-fit (GoF), and multiple indices of different types should be used [33]. We assessed the GoF with the normed Chi-square (χ^2^/df), the root mean square error of approximation (RMSEA), and standardized root mean residual (SRMR) from the class of absolute indices, and the comparative fit index (CFI), Normed fit index (NFI) and Tucker-lewis index (TLI) from the class of the incremental indices. The cut-off values were acceptable for our data: ratio 3:1 or less for *χ*^2^/df, 0.06 or less for RMSEA, 0.08 or less for SRMR, and > 0.90 for CFI, NFI, and TLI [32].

Based on the findings of the CFA, the convergent validity and discriminant validity covering 32 items across 10 dimensions were assessed to demonstrate the construct validity further. The convergent validity was determined by the average variance extracted (AVE) and composite reliability (CR), and was deemed acceptable if the values of AVE and CR were > 0.5 and 0.7, respectively [34]. Discriminant validity assesses whether the items in a dimension are strongly correlated with another dimension, and it was acceptable if correlation coefficient < Sqrt (AVE) [24, 35]. Moreover, Spearman correlation coefficients were calculated to examine the correlation between the 10 dimensions of the Chinese version of HSOPS 2.0 and the single item that measured *patient safety grade* [36].

#### Stage 3: The testing of reliability

Cronbach’s alpha coefficient and McDonald’s omega were used to evaluate the reliability of the overall questionnaire and each dimension, with the value > 0.7 indicating an acceptable reliability [37, 38]. Test-retest reliability was assessed using the intraclass correlation coefficient (ICC) (ICC < 0.4, poor; 0.4 ~ 0.59, average; 0.6 ~ 0.74, acceptable; > 0.75, good) [39]. To calculate ICC, the “two-way mixed effects” model, “single measurement” type, and “absolute agreement” were used [40].

## Result

### Sample and response statistics

A total of 1013 valid questionnaires were included and the response rate was 78.0%. 897 (88.6%) nurses were female, 886 (87.5%) were aged from 21 to 44 years old, 736 (72.7%) had a bachelor and higher degree in nursing. The majority of nurses usually worked more than 40 hours per week (86.5%) and dealt with patients directly (94.1%) (Table 2).

**Table 2:** Demographic Characteristics of Participants (*N* = 1013)

Characteristic		Number	Percent
Gender	Male	116	11.5
	Female	897	88.6
Age	Less than 21 years old	8	0.8
	21 to 34 years old	637	62.9
	35 to 44 years old	249	24.6
	45 to 54 years old	108	10.7
	More than 54 years old	11	1.1
Education	Associate degree	277	27.3
	Bachelor’ s degree	587	58.0
	Master’ s degree and above	149	14.7
Positional titles	Junior and below	698	68.9
	Middle	244	24.1
	Senior	71	7.0
Direct interaction with patients	Yes	953	94.1
	No	60	5.9
Employment type	Permanent, full-time	653	64.5
	Temporary, full-time	360	35.5
Years in nursing	Less than 1 year	90	8.9
	1 to 5 years	361	35.6
	6 to 10 years	292	28.8
	11 to 15 years	124	12.2
	16 to 20 years	71	7.0
	21 or more years	75	7.4
Years in current hospital	Less than 1 year	82	8.1
	1 to 5 years	345	34.1
	6 to 10 years	301	29.7
	11 or more years	285	28.1
Years in current unit	Less than 1 year	158	15.6
	1 to 5 years	434	42.8
	6 to 10 years	239	23.6
	11 or more years	182	18.0
Hours worked per week in hospital	Less than 30 hours per week	10	1.0
	30 to 40 hours per week	127	12.5
	More than 40 hours	876	86.5

Table 3 shows the descriptive statistics for the Chinese version of HSOPS 2.0. The three dimensions with the highest positive response rates were *Teamwork* (93.0%), *Supervisor, Manager, or Clinical Leader Support for Patient Safety* (91.0%), and *Communication About Error* (87.0%). The three dimensions with lowest positive response rate were *Response to Error* (44.0%), *Reporting Patient Safety Events* (46.0%), and *Staffing and Work pace* (51.0%). The item C2, i.e., “when errors happen in this unit, we discuss ways to prevent them from happening again.” (99.0%) had the highest positive response rate, while item A10, i.e., “when staff make errors, this unit focuses on learning rather than blaming others.” the lowest positive response rate (22.0%).

**Table 3:** Positive response rate of each item and Cronbach’s α for dimensions

Dimension/items (internal consistency and test-retest reliability coefficient)		Internal consistency (*N* = 1013, Cronbach’s α)		Test-retest Reliability (*n* = 200, ICC)	McDonald’s hierarchical dimensions omega(ω)	M ± SD	Positive responses rate (PPRs)	
		US	China				US	China
DD1.	Teamwork	0.76	0.75	0.95，p<0.001	0.86	4.39 ± 0.60	82.0	93.0
A1.	In this unit, we work together as an effective team.						88.0	95.0
A8.	During busy times, staff in this unit help each other.						87.0	94.0
A9r.	There is a problem with disrespectful behavior by those working in this unit.						70.0	91.0
DD2.	Staffing and Work Pace	0.67	0.75	0.87，p<0.001	0.84	3.21 ± 0.92	58.0	51.0
A2.	In this unit, we have enough staff to handle the workload.						53.0	52.0
A3r.	Staff in this unit work longer hours than is best for patient care.						54.0	30.0
A5ra.	This unit relies too much on temporary staff.						62.0	57.0
A11r.	The work pace in this unit is so rushed that it negatively affects patient safety.						61.0	65.0
DD3.	Organizational Learning – Continuous Improvement	0.76	0.87	0.78，p<0.001	0.92	3.71 ± 0.96	72.0	61.0
A4.	This unit regularly reviews work processes to determine if changes are needed to improve patient safety.						74.0	61.0
A12.	In this unit, changes to improve patient safety are evaluated to see how well they worked.						68.0	64.0
A14r.	This unit lets the same patient safety problems keep happening.						74.0	58.0
DD4.	Response to Error	0.83	0.82	0.92，p<0.001	0.89	3.07 ± 0.94	64.0	44.0
A6r.	In this unit, staff feel like their mistakes are held against them.						71.0	40.0
A7r.	When an event is reported in this unit, it feels like the person is being written up, not the problem.						62.0	59.0
A10.	When staff make errors, this unit focuses on learning rather than blaming individuals.						58.0	22.0
A13r.	In this unit, there is a lack of support for staff involved in patient safety errors.						65.0	53.0
DD5b.	Supervisor or Clinical Leader Support for Patient Safety	0.77	0.68	0.85，p<0.001	0.84	4.24 ± 0.53	80.0	91.0
B1b.	My supervisor or clinical leader seriously considers staff suggestions for improving patient safety.						79.0	92.0
B2rb.	My supervisor or clinical leader wants us to work faster during busy times, even if it means taking shortcuts.						84.0	87.0
B3b.	My supervisor or clinical leader takes action to address patient safety concerns that are brought to their attention.						78.0	95.0
DD6.	Communication About Error	0.89	0.83	0.80，p<0.001	0.96	4.38 ± 0.73	71.0	87.0
C1.	We are informed about errors that happen in this unit.						70.0	98.0
C2.	When errors happen in this unit, we discuss ways to prevent them from happening again.						74.0	99.0
C3.	In this unit, we are informed about changes that are made based on event reports.						69.0	63.0
DD7.	Communication Openness	0.83	0.75	0.78，p<0.001	0.84	3.09 ± 0.92	75.0	51.7
C4.	In this unit, staff speak up if they see something that may negatively affect patient care.						83.0	59.0
C5.	When staff in this unit see someone with more authority doing something unsafe for patients, they speak up.						72.0	56.0
C6.	When staff in this unit speak up, those with more authority are open to their patient safety concerns.						75.0	55.0
C7r.	In this unit, staff are afraid to ask questions when something does not seem right						71.0	36.6
DD8.	Reporting Patient Safety Events	0.75	0.82	0.90，p<0.001	0.92	3.45 ± 1.13	74.0	46.0
D1.	When a mistake is caught and corrected before reaching the patient, how often is this reported?						65.0	44.0
D2.	When a mistake reaches the patient and could have harmed the patient, but did not, how often is this reported?						83.0	48.0
DD9.	Hospital Management Support for Patient Safety	0.77	0.87	0.78，p<0.001	0.92	4.09 ± 0.80	67.0	80.0
F1.	The actions of hospital management show that patient safety is a top priority.						79.0	86.0
F2.	Hospital management provides adequate resources to improve patient safety.						73.0	86.0
F3r.	Hospital management seems interested in patient safety only after an adverse event happens.						49.0	67.0
DD10.	Handoffs and Information Exchange	0.72	0.93	0.84，p<0.001	0.96	3.71 ± 1.06	64.0	72.0
F4r.	When transferring patients from one unit to another, important information is often left out.						73.0	73.0
F5r.	During shift changes, important patient care information is often left out.						56.0	73.0
F6.	During shift changes, there is adequate time to exchange all key patient care information.						63.0	69.0

r: negatively worded item; a: float or PRN staff of item A5 was deleted; b: manager of dimension DD5 was deleted

Compared to the results from the original U.S. data in 2021, the overall average positive response rate for dimensions was 67.7% in this study vs. 70.7% in the U.S. study. The dimensions related to *Teamwork* (82.0%) and *Supervisor, Manager, or Clinical Leader Support for Patient Safety* (80.0%) had the highest rate in both U.S. and this study. The dimensions *Staffing and Work Pace* (58.0%) had the lowest positive rate in U.S. study, while it was *Response to Error* (44.0%), and *Reporting Patient Safety Events* (46.0%) (Table 3) in the present study.

### The content validity of the questionnaire

Two rounds of expert consultations were used to modify the questionnaire and evaluate its content validity, and the recovery rates of the two rounds were 100%. The value of Cs was 0.76, the Ca was 0.90, and the authoritative coefficient of this study was 0.87 (> 0.70), with a good authoritative degree. The final Chinese version of HSOPS 2.0 comprised 34 items across 10 dimensions, same as the original HSOPS 2.0. Yet, experts adjusted for some words that did not adapt to the health care system of China. The ‘float or PRN staff’ part was deleted from item A5. Also, the word ‘manager’ was deleted from the dimension of *Supervisor, Manager, or Clinical Leader Support for Patient Safety* because such kinds of categorizations are uncommon in Chinese hospitals. Finally, the I-CVI scores ranged from 0.84 to 1.00, and the S-CVI was 0.98, providing evidence of strong content validity.

### The readability and understandability of the questionnaire

Respondents completed the initial draft questionnaire within 6–15 minutes. The questionnaire was considered not long and easy to complete. All items were considered relevant to each dimension, and no suggestions were made regarding excluding any items or adding new items. Unclear expression statements of some items were revised in case of misunderstanding.

### The construct validity of the questionnaire

The KMO test (0.913) and Bartlett’s test of Sphericity (*p* < 0.001) indicated our data were suitable for factor analysis [41].

CFA was applied to determine the construct validity of the questionnaire, and revealed acceptable or excellent values for absolute indices: χ^2^/df = 4.05, RMSEA = 0.06, SRMR = 0.05 and acceptable for the incremental index CFI = 0.94, NFI = 0.93, TLI = 0.93 (Table 4). All items within each dimension had acceptable factor loadings > 0.4, ranging from 0.41 to 0.97. A slightly high correlation was found between dimensions “*Organizational Learning - Continuous Improvement*” and “*Communication About Error Hand offs*” (0.83) and “*Information Exchange*” (0.73) (Additional file 1: Supplementary fig. 1).

**Table 4:** Fit indices for the Chinese version of HSOPS 2.0

Fit indices		Threshold	Interpretation
CFI	0.94	(> 0.90)	Acceptable
NFI	0.93	(> 0.90)	Acceptable
TLI	0.93	(> 0.90)	Acceptable
RMSEA	0.06	(< 0.06)	Excellent
SRMR	0.05	(< 0.08)	Excellent
χ2 /df	4.05	(< 5)	Acceptable

*GFI/CFI/NFI/TLI* Terrible (< 0.09) Acceptable(< 0.95)；Excellent(> 0.95)

*RMSEA* Terrible (> 0.08);Acceptable (> 0.06); Excellent(< 0.06)

*SRMR* Terrible (> 0.10) Acceptable (> 0.08); Excellent(< 0.08)

*χ2 /df* Terrible (> 5) Acceptable (> 3); Excellent(> 1)

Table 5 shows each dimension’s value of the AVE and CR for convergent validity. The square root of every AVE value and correlation coefficients between dimensions are shown in Table 6. Study findings showed that the values of CR were > 0.70, indicating that the combined reliability is good; the values of AVE of 10 dimensions were distributed between 0.51 and 0.83 (> 0.50), showing suitable convergent validity. The Sqrt (AVE) were higher than the correlation coefficient for each of the two dimensions, which suggested acceptable discriminant validity.

**Table 5:** Convergent validity and composite reliability for dimensions

Dimension		Factor loading	AVE	CR
DD1	Teamwork	0.86, 0.79, 0.51	0.54	0.77
DD2	Staffing and Work Pace	0.75, 0.63, 0.90, 0.53	0.51	0.80
DD3	Organizational Learning – Continuous Improvement	0.89, 0.863, 0.749	0.70	0.87
DD4	Response to Error	0.76, 0.82, 0.42, 0.88	0.55	0.82
DD5	Supervisor or Clinical Leader Support for Patient Safety	0.87, 0.41, 0.84	0.54	0.77
DD6	Communication About Error	0.82, 0.92, 0.97	0.82	0.93
DD7	Communication Openness	0.83, 0.85, 0.71, 0.42	0.52	0.81
DD8	Reporting Patient Safety Events	0.93, 0.68	0.67	0.80
DD9	Hospital Management Support for Patient Safety	0.80, 0.92, 0.94	0.79	0.92
DD10	Handoffs and Information Exchange	0.96, 0.92, 0.84	0.83	0.93

**Table 6:** Discriminant validity

Dimension	DD1	DD2	DD3	DD4	DD5	DD6	DD7	DD8	DD9	DD10
DD1	0.54									
DD2	0.32	0.51								
DD3	0.39	0.67	0.70							
DD4	0.29	0.46	0.54	0.55						
DD5	0.49	0.33	0.41	0.25	0.54					
DD6	0.29	0.69	0.83	0.45	0.28	0.82				
DD7	0.19	0.62	0.65	0.35	0.25	0.68	0.52			
DD8	0.13	0.35	0.38	0.16	0.16	0.38	0.33	0.67		
DD9	0.20	0.41	0.49	0.29	0.25	0.44	0.37	0.19	0.79	
DD10	0.31	0.62	0.73	0.44	0.42	0.67	0.57	0.36	0.45	0.83
The square roots of AVE	0.74	0.72	0.83	0.74	0.74	0.91	0.72	0.82	0.89	0.91

Also, the 10 dimensions were positively correlated with *patient safety grade*, with correlations ranging from 0.10 to 0.41, providing further evidence of the construct validity of the Chinese version of HSOPS 2.0.

### The reliability of the questionnaire

The Cronbach’s α of the overall questionnaire was 0.92. and of the dimensions ranged from 0.68 to 0.93. Except dimension “Supervisor or Clinical Leader Support for Patient Safety” (*α* = 0.68), the Cronbach’s α of other dimensions were above 0.70. The omega values of all dimensions were above 0.70 and the total score was 0.93. The test-retest reliability was 0.83 for the overall score, with each dimension ICC ranging from 0.78 to 0.95. The results showed acceptable reliability (Table 3).

## Discussion

To the best of our knowledge, this is the first study that performed a cross-cultural adaptation and psychometric property testing of the Chinese version of HSOPS 2.0 following rigorous guidelines. Overall, the Chinese version of HSOPS 2.0 showed good validity and adequate reliability. This data may facilitate future research and clinical practice related to PSC.

Firstly, in order to make the questionnaire more suitable for the Chinese healthcare system, we deleted words ‘float or PRN staff’ of item A5 (*This unit relies too much on temporary, float, or PRN staff.*) because such kinds of categorizations are uncommon in China. Similarly, the other Asian countries such as Japan [42] and Korea [43] do not have many float or PRN nurses, although they 7are developed countries. In the Korean version of HSOPS 2.0, the researchers removed the whole item A5 because they thought nurses (including temporary staff) have fixed-term contracts (e.g., 1 year of full time work) [43]. However, in this study, we kept the term “temporary staff” because most Chinese people feel that if they cannot have a permanent contract job, they will consider themselves as temporary staff (including contract-based nurses or‘Bianwai nurses’who are often receive low wages, and no or reduced benefits, at the discretion of employing hospitals [44]). They could create unsettling feelings and job insecurity [45] that affect their work attitudes and behaviors [46, 47]. Meanwhile, we believe that temporary staff of the original HSOSP 2.0 was designed to measure the negative effects on patient safety resulting from the attitudes and behaviors of these temporary employees, in line with the Chinese HSOPS 1.0 [18]. The interviewees and the expert panel believe that keeping the new item A5 (This unit relies too much on temporariness) in the Chinese version of HSOPS 2.0 can help to understand the culture of patient safety in Chinese hospitals. In addition, the word ‘manager’ was deleted from *Supervisor, Manager, or Clinical Leader Support for Patient Safety* dimension as it is not common in Chinese hospitals. Also, the I-CVIs and the S-CVI were considered excellent, providing the evidence of strong content validity and supporting the deletion of words “float or PRN staff” and “manager” from the Chinese version of HSOPS 2.0.

According to the results of CFA, this study confirmed the 10-factor structure of the Chinese version of HSOPS 2.0. The factor loading was all over 0.40, consistent with the original HSOPS 2.0 [48] which demonstrated a good construct validity. In addition, all the dimensions were related to *patient safety grade*, providing further evidence of the construct validity of the Chinese version of HSOPS 2.0. This finding is consistent with the Korean version of HSOPS 2.0 [43].

The Chinese version of HSOPS 2.0 demonstrated adequate reliability. Internal consistency reflects the interrelatedness of the items. In this study, the internal consistency coefficients of dimensions ranged from 0.68 to 0.93. The Cronbach’s α value of the dimension “*Supervisor or Clinical Leader Support for Patient Safety*” was 0.68, which is consistent with data provided by the U.S. (0.77) and Korea (0.75) [43, 48]. Although the value for this dimension in the Chinese version of HSOPS 2.0 did not reach the 0.70 threshold recommended by Nunali and Bernstein [49], some data suggest that Cronbach′s α coefficient > 0.6 is still acceptable [40]. In addition, the Cronbach’s α value was as similarly low as the one for the same dimension of the Chinese HSOPS 1.0 (0.51), which may be explained by a diverse culture of organizational leadership, policy beliefs, and management patterns between the U.S., Korea, and China [17]. The small number of items (*n* = 3) may also explain the relatively low internal consistency. Going further than Cronbach’s alpha, the McDonald’s omega values of all the dimensions and overall questionnaire showed a good reliability [38]. Test-retest reliability reflects the consistency of a measure across time. In this study, we used a month interval to assess the PSC and re-test the data assuming that the PSC of the dimension remained stable during this interval. For the new measurement tool development, it is believed that the re-test reliability needs to be more than 0.7. In this study, the ICC ranged from 0.78 to 0.95, suggesting that the retest reliability of the questionnaire was up to the requirements of psychological measurement, showing good stability. Overall, the above evidence reveals that the Chinese version of HSOPS 2.0. had adequate reliability [36].

Finally, we found that the overall positive response rate for the Chinese version of HSOPS 2.0 was in line with that of the U.S. and superior to the Chinese HSOPS 1.0 [18, 50]. The “*Teamwork Supervisor, or Clinical Leader Support for Patient Safety*” and “*Communication About Error*” had the highest positive response rates, in fact much higher than the U. S. findings on the original HSOPS 2.0, which was consistent with the studies using the Chinese HSOPS 1.0 for remaining dimensions as areas of strength [18, 51, 52]. This result could be explained by the following reasons: (1) AHRQ mentioned that the HSOPS 2.0 might get higher scores due to the modification of both positive words and negative words based on the 2017 and 2019 pilot tests [19]; (2) increasing attention has been paid to PSC in China, and some significant progress has been made since PSC was included in the China National Patient Safety Goals in 2015. In addition, this survey was conducted in Shanghai, which has a relatively developed medical management system and strong awareness of PSC and possesses a special patient safety management department resulting in advanced PSC.

The positive response rates of “*Response to Error*”, “*Communication Openness*” and “*Reporting Patient Safety Events*” were much lower in the present study than U.S. study, especially regarding A6, A10, C7, D1, and D2 sections. This may be related to a developing adverse event reporting system, complicated process and lack of support, among which non-punitive culture remains the biggest challenge in Chinese hospitals. Even though some hospitals encourage reporting adverse events, many staff still worry that adverse events may affect their promotion. In other words, plenty of healthcare workers still do not have confidence in the non-punitive policy regarding error reporting, and they perceive ‘penalty’ to be the greatest barrier to encouraging the reporting of errors as before [17]. This might also explain low scores on “*Communication Openness*” and “*Response to Error*” [13, 53]. A non-punitive culture is exactly what Chinese hospital management lacks and needs to be improved for patient safety and high quality health care.

The positive response rate of *Staffing and Work Pace* in this study was 51.0%, which is slightly lower than that in the U.S. (58.0%), while notable difference was observed in the positive response rate of A3, i.e., “Staff in this unit work longer hours than is best for patient care.” Insufficient human allocation and the fast pace of work in health care are still common problems numerous countries are faced with [54], where more convenient measures are needed to improve the situation [55, 56]. In 2017, the China Care Quality Report showed that the overall median nurse-to-patient (NTP) ratio at large general hospitals improved from 1:11.2 to 1:10 from 2014 to 2016 [57]. Moreover, a study from 2016 showed that the NTP ratio in large general hospitals in China was 1:8.0 [58]. but the nurse workload was still significantly higher than the minimum NTP ratios of 1:4 and 1:5 for general medical and surgical wards set by the State of California in 2004 [59].

Staffing is also important for improving patient safety and quality of care. The National Nursing Career Development Plan (2021–2025) formulated by the Chinese National Health Commission stipulated a plan to increase the total number of nurses in China to 5.5 million (vs. 4.7 million in 2020), the number of registered nurses per 1000 population to 3.8 (vs.3.3 in 2020), and the ratio of nurses in hospital units to actual open beds to 0.9:1 (vs. 0.6:1 in 2020) [60]. Therefore, substantial efforts should be made to alleviate the shortage and unequal distribution of nurses.

## Limitations

This study has several limitations. Firstly, the respondents were recruited using convenience sampling. Secondly, participants were only nurses, while other staff were not included. Thirdly, the sample collection of this study only covered the Shanghai area. Taking the above into consideration, the results might not represent all Chinese nurses and cannot be generalized to other healthcare professionals. Therefore, future studies of the Chinese version of HSOPS 2.0 will have to ensure the representativeness of study samples from diverse provinces and regions. In addition, the concurrent validity was not evaluated because there is not an appropriate scale which was recommended as the gold standard to evaluate patient safety culture.

## Conclusions

This study investigated the psychometric properties of HSOPSC 2.0 in a Chinese healthcare context. The Chinese version of HSOPS 2.0 demonstrated good validity and adequate reliability (including internal consistency and test-retest reliability) in a Chinese sample of hospital nurses, which suggests that it can be used to measure nurse-perceived patient safety culture in future research and practice. Psychometric properties of the Chinese version of HSOPS 2.0 among other Chinese healthcare professionals remain to be confirmed, and more research is needed to investigate its psychometric properties within a broader validation context.

## Supplementary Information

**Additional file 1.**

## Data Availability

The datasets generated and/or analyzed during the current study are not publicly available due confidentiality of the participants but are available from the corresponding author upon reasonable request.

## Abbreviations

HSOPS: Surveys on Patient Safety Culture™ (SOPS®) Hospital Survey
PSC: Patient safety culture
PS: Patient safety
