# Supplementary material for: Cross-cultural adaptation and validation of the Chinese version of the revised surveys on patient safety culture™ (SOPS®) hospital survey 2.0
Source: BMC Nurs. 2022 Dec 26;21:369. doi: 10.1186/s12912-022-01142-3 (PMC9792160; doi:10.1186/s12912-022-01142-3)
Supplement: Supplementary file 1 — Additional file 1. [file 12912_2022_1142_MOESM1_ESM.pdf]

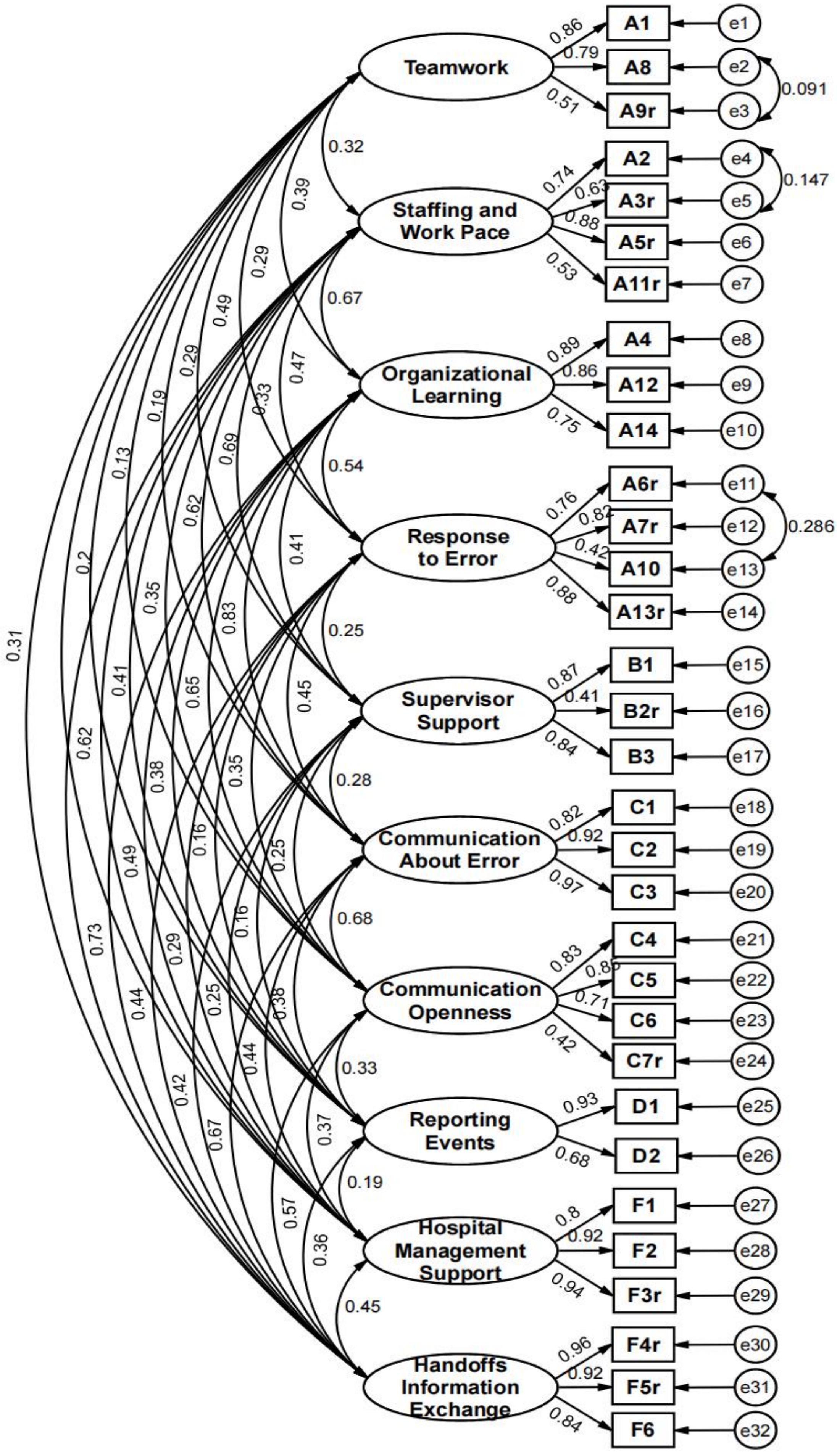

Supplementary figure 1 Model for CFA of the Chinese HSOPS 2.0. All items within each dimension had acceptable factor loadings > 0.4, ranging from 0.41 to 0.97. A slightly high correlation was found between dimensions "*Organizational Learning - Continuous Improvement*" and "*Communication About Error Hand offs*" (0.83) and "*Information Exchange*" (0.73)
